# IL-1β Is Upregulated in the Diabetic Retina and Retinal Vessels: Cell-Specific Effect of High Glucose and IL-1β Autostimulation

**DOI:** 10.1371/journal.pone.0036949

**Published:** 2012-05-16

**Authors:** Yang Liu, Montserrat Biarnés Costa, Chiara Gerhardinger

**Affiliations:** Schepens Eye Research Institute and the Department of Ophthalmology, Harvard Medical School, Boston, Massachusetts, United States of America; University of Bremen, Germany

## Abstract

Many molecular and cellular abnormalities detected in the diabetic retina support a role for IL-1β-driven neuroinflammation in the pathogenesis of diabetic retinopathy. IL-1β is well known for its role in the induction and, through autostimulation, amplification of neuroinflammation. Upregulation of IL-1β has been consistently detected in the diabetic retina; however, the mechanisms and cellular source of IL-1β overexpression are poorly understood. The aim of this study was to investigate the effect of high glucose and IL-1β itself on IL-1β expression in microglial, macroglial (astrocytes and Müller cells) and retinal vascular endothelial cells; and to study the effect of diabetes on the expression of IL-1β in isolated retinal vessels and on the temporal pattern of IL-1β upregulation and glial reactivity in the retina of streptozotocin-diabetic rats. IL-1β was quantified by RealTime RT-PCR and ELISA, glial fibrillar acidic protein, α2-macroglobulin, and ceruloplasmin by immunoblotting. We found that high glucose induced a 3-fold increase of IL-1β expression in retinal endothelial cells but not in macroglia and microglia. IL-1β induced its own synthesis in endothelial and macroglial cells but not in microglia. In retinal endothelial cells, the high glucose-induced IL-1β overexpression was prevented by calphostin C, a protein kinase C inhibitor. The retinal vessels of diabetic rats showed increased IL-1β expression as compared to non-diabetic rats. Retinal expression of IL-1β increased early after the induction of diabetes, continued to increase with progression of the disease, and was temporally associated with upregulation of markers of glial activation. These findings point to hyperglycemia as the trigger and to the endothelium as the origin of the initial retinal upregulation of IL-1β in diabetes; and to IL-1β itself, via autostimulation in endothelial and macroglial cells, as the mechanism of sustained IL-1β overexpression. Interrupting the vicious circle triggered by IL-1β autostimulation could limit the progression of diabetic retinopathy.

## Introduction

Despite the improvements in the management of hyperglycemia and hypertension, most adult with diabetes do not achieve the target levels of glycemic and blood pressure control [1], [2]. For these reasons, diabetic retinopathy remains a prevalent complication of diabetes and a leading cause of vision loss and blindness in the adult population [3]. In order to develop effective treatments to prevent vision loss in diabetes, there is a need for a better understanding of the mechanisms linking the diabetic milieu to retinal damage.

Growing evidence suggest that IL-1β-driven neuroinflammation could be one such mechanism. Although diabetic retinopathy does not have the characteristics of an overt inflammatory reaction, features of neuroinflammation are present in the diabetic retina [4]. These include macroglial and microglial activation, leukostasis, increased vascular permeability, and increased expression of cytokines, acute phase proteins, and vasoactive peptides [5]–[11].

IL-1β is a multifunctional proinflammatory cytokine [12] and the main trigger of the neuroinflammatory cascade [13]. Release of IL-1β is one of the earliest events after traumatic and ischemic brain injures as well as in chronic neurodegenerative diseases. Once released, IL-1β elicits a multitude of effects on target cells that lead to and modulate the inflammatory response and tissue damage [13]. By inducing its own synthesis via autocrine/paracrine autostimulation IL-1β is not only a trigger but also an amplifier of inflammation [14]–[18].

Several observations support a role for IL-1β as a probable mediator of retinal damage in diabetes. IL-1β is increased in the retina in experimental diabetes [7], [10], [19], [20], and such increase is due to increased retinal synthesis [10]. Interleukin converting enzyme/caspase-1, the enzyme responsible for the production of biological active IL-1β [12], is activated in the retina in both human and experimental diabetic retinopathy [21], [22]. Intercellular adhesion molecule-1, the transcription factors CEBP-β and -δ, and other IL-1β-dependent genes are upregulated in the diabetic retina [10], [23]. Mice lacking IL-1 receptor I are protected from the development of acellular capillaries in diabetes [22].

Monocytes, aortic and retinal endothelial cells, and retinal Müller cells have been reported to upregulate and/or secrete increased amount of IL-1β when exposed to high glucose in vitro [24]–[28]. Although these findings point to vascular endothelial cells and Müller cells as a possible source of increased IL-1β in the diabetic retina, there is no evidence that vascular cells or Müller cells overexpress IL-1β in diabetes. Moreover, the contribution of microglial cells and astrocytes as a source of IL-1β and the mechanism of IL-1β upregulation in the diabetic retina remain unknown.

To identify the cellular source and mechanism of the diabetes-induced upregulation of IL-1β in the retina we studied the effect of high glucose and IL-1β itself on the expression of the cytokine in microglial, macroglial, and retinal vascular endothelial cells; and the effect of diabetes on the expression of IL-1β in isolated retinal vessels and on the temporal pattern of IL-1β upregulation and glial reactivity in the retina of streptozotocin-diabetic rats.

## Methods

### Ethical Statement

All procedures involving animals conformed to the Association for Research in Vision and Ophthalmology resolution on the use of animals in research and were approved by the Animal Use and Care Committee of the Schepens Eye Research Institute (Protocols # S-163-0309 and S-189-0411).

### Cell isolation and culture

Rat microglial cells (RMG) and astrocytes (RA) were isolated from 1–3 days old Wistar rats as described by Tham et al. [29] and Ramsauer et al. [30] respectively. Cerebral cortices were dissociated by repeated pipetting in ice-cold calcium-free Hanks balanced saline solution and filtered through a 100 µm nylon mesh. The dissociated cells were pelleted by centrifugation at 1400 rpm for 5 min, resuspended in 10% FBS-DMEM, and plated on poly-L-lysine-coated tissue flasks. The medium was replaced on days 3, 5 and 9 after plating. For isolation of RMG, at day 13 the flasks were shaken at 100 rpm for 2 h at 37°C. The supernatant was collected and the detached microglial cells were pelleted by centrifugation at 350×g for 5 min, resuspended in 10% FBS-DMEM, and plated on poly-L-lysine-coated dishes. For isolation of RA, at day 10 the flasks were shaken at 200 rpm at 37°C for 2 days and at 100 rpm for 2 additional days. After washing with PBS, the adherent cell monolayers were trypsinized and the cells plated on poly-L-lysine coated dishes and cultured in 10% FBS-DMEM. Purity of primary isolates and subcultures was assessed by CD11b immunostaining and isolectin B4 binding for microglia and glial fibrillary acidic protein (GFAP) immunostaining for astrocytes (Fig. S1). Rat retinal Müller cells (RMC) were isolated from 6 days old Wistar rats (Taconic Farms, Hudson, NY) as described by Hicks et al. [31]. After an overnight at room temperature in the dark in DMEM (Gibco, Life Technologies, Carlsbad, CA) the enucleated eyes were incubated at 37°C for 30 min in the same medium supplemented with 0.1% trypsin and 70 U/ml collagenase (Sigma, St. Louis, MO). The retinas were dissected, dissociated by repeated pipetting, and cultured in DMEM supplemented with 10% FBS for 6 days before the first medium change. Purity of primary isolates and subcultures was assessed by vimentin and glutamine synthetase immunostaining (Fig. S1). Bovine retina endothelial cells (BREC) were isolated and cultured using procedures optimized to prevent pericyte contamination as previously described [32]. For this purpose, the primary cultures were subjected to brief trypsinization (90 s at room temperature) and the detached cells were replated on fibronectin-coated dishes and cultured in endothelial growth medium (EBM supplemented with bovine brain extract 1∶1000, Lonza, Walkersville, MD) containing 10% horse serum from platelet-poor plasma (Sigma) [32]. Purity of primary isolates and subcultures was assessed by von Willebrand factor immunostaining (Fig. S1).

Experiments with RMG were performed with freshly isolated primary cells, those with RA and RMC with cells at passage 2 to 5, and those with BREC with cells at passage 4 to 7. All cells were maintained at 37°C in 5% CO2, 95% air, and media were changed every 2 days. To study the effect of high glucose, cells were exposed to either normal glucose (5.5 mM, NG) or high glucose (25 mM, HG) concentrations for 4 days. Cell cultured in equimolar mannitol and, in some experiments, L-glucose were used as control for the effects of hypertonicity. To study the effect of PKC activation, BREC, grown to confluency in NG medium, were stimulated with 10 and 30 nM phorbol 12 myristate 13-acetate (PMA, Sigma) for 4 hours before harvesting [33]. For the PKC inhibition experiments, BREC were cultured for 4 days in NG, HG, and HG containing the non-selective PKC inhibitor calphostin C (50 nM, EMD-Calbiochem, Gibbstown, NJ). To test for the effect of IL-1β autostimulation, cells were cultured in NG until confluency and then incubated in serum-free NG medium for 1 day before stimulation with recombinant IL-1β (10 ng/ml medium) for 4 hours. Bovine IL-1β (Thermo Scientific, Rockford, IL) was used in the experiments with BREC, human recombinant IL-1β (R&D, Minneapolis, MN) was used in the experiments with RMC, RA, and RMG. In some experiments, RMG pre-incubated in serum-free medium as above, were stimulated with LPS at 1 µg/ml medium (from E. Coli, Sigma).

### Animals

Sprague-Dawley male rats (5 weeks of age; Taconic Farms) were randomly assigned to a diabetic and a control group. Induction of diabetes with streptozotocin (55 mg/kg body weight) and treatment of diabetic rats with maintenance insulin were as described [10]. Diabetic rats and age-matched non-diabetic control rats were studied at different time points from the induction of diabetes (from 1.5 to 5 months). Rats assigned to quantification of IL1β – RNA and protein (ELISA) – were killed by CO_2_ inhalation, the eyes immediately removed and the retinas dissected and processed for the isolation of whole retina RNA and proteins or for the isolation of retinal vessels. To avoid artifact due to plasma α2-macroglobulin and ceruloplasmin, rats assigned to the isolation of retinal proteins for western blotting were perfused through the left ventricle with phosphate buffered saline under deep anesthesia as previously described [10]. To assess the severity of diabetes, blood was collected at the time of death by cardiac puncture for the measurement of glycated hemoglobin (A1C) as previously described [10]. For the group with the shorter duration of diabetes (1.5 months) severity of diabetes was assessed by measuring blood glucose levels. The characteristics of the diabetic and control rats are summarized in table 1.

**Table 1:** Characteristics of study rats.

Study duration	group	n	body weight (g)	glycemia (mg/dl)	A1C (%)
1.5 months	diabetes	8	308±20*	633±138*	—
	control	8	492±42	206±100	—
2.5–3 months	diabetes	19	382±30*	—	15.7±2.6*
	control	19	561±69	—	5.8±1.0
4.5–5 months	diabetes	27	401±71*	—	16.0±1.8*
	control	27	605±63	—	6.1±1.0

* *P*<0.0001 vs control.

### Isolation of retinal vessels

Rat retinal vessels were isolated by hypotonic lysis of the fresh retina as previously described [34], [35]. As previously documented, this method yields the retinal vascular network free of glia and neural contamination [35].

### RNA isolation and RealTime RT-PCR analysis

Total RNA from cultured cells, whole retinas, and retinal vessels, was isolated using the RNeasy mini kit (Qiagen, Valencia, CA) following the on-column DNase digestion protocol [34]. Relative expression of IL-1β and IL1R1 in cultured cells, IL-1β in retinal vessels, and IL-1β, IL-6, and TNF-α in whole retinas was determined by RealTime RT-PCR using the comparative ΔΔCt method as described [10], [34]. Primers and probe sets for bovine IL-1β and β-actin are listed in Table S1, those for rat IL-1β, IL1R1, IL-6, TNF-α, and β-actin were from Applied Biosystems (Life Technologies, Carlsbad, CA).

### Immunoblotting and ELISA

Preparation of retinal protein lysates and immunoblotting for α2-macroglobulin, ceruloplasmin, GFAP, and β-actin were performed as described [10], [23], [34]. IL-1β protein levels were quantified by colorimetric sandwich ELISA (Rat IL1β Quantikine, R&D Systems, Minneapolis, MN). Each samples was assayed in duplicate using 50 µg of total protein per well.

### Statistical Analysis

Data are summarized as means ± SD and analyzed by unpaired Student's t test for two groups comparison or ANOVA followed by Fisher's protected least significant differences test for multiple groups comparisons. Analysis of the PMA dose response effect (p for trend) was performed by linear regression using IL-1β as the dependent variable and the PMA concentration as the independent variable. Statistical analysis was performed with StatView 5.0 (SAS Institute, Cary, NC). A *P* value≤0.05 was considered as evidence of a statistically significant difference between groups.

## Results

### HG induces IL-1β expression in retinal vascular endothelial cells but not in Müller cells, astrocytes, or microglia

**Figure 1:**
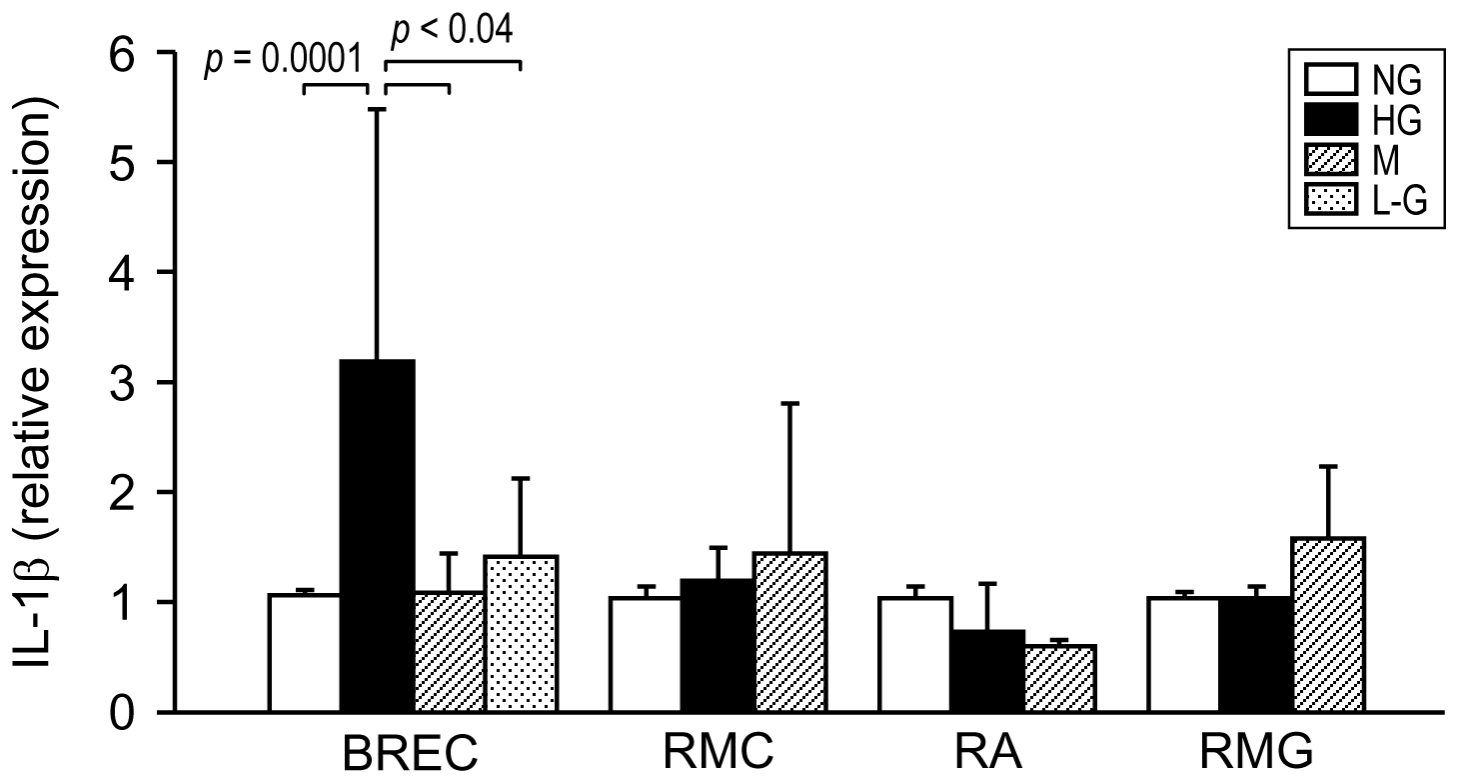
Effect of high glucose on IL-1β expression in retinal endothelial, macroglial, and microglial cells. Confluent culture of bovine retinal vascular endothelial cells (BREC), rat Müller cells (RMC), astrocytes (RA), and microglia (RMG) were exposed to normal glucose (5.5 mM, NG) or high glucose (25 mM glucose, HG) for 4 days. Equimolar mannitol (M) and L-glucose (L-G) (BREC only) were used as osmolar control. Total RNA was extracted and IL-1β mRNA levels were assayed by quantitative RealTime RT-PCR. Relative expression of IL-1β mRNA was calculated by the comparative C_T_ method using β-actin as endogenous control (ΔΔC_T_ method). Bars represent mean ± SD of the results obtained in independent experiments form 5 isolates of BREC (each tested in multiple experiments comparing NG, HG, and M. L-G was tested in three experiments, each using a different isolate), and 3 isolates of RMC, RA, and RMG. BREC: NG, HG, and M, n = 14, L-G, n = 3; RMC: NG, HG, and M, n = 4; RA: NG and HG, n = 5, M n = 2; RMG: NG and HG, n = 4, M n = 2.

Retinal vascular endothelial and Müller cells have been shown to upregulate IL-1β when exposed to high glucose concentration. To test whether high glucose has a similar effect also on astrocytes and microglia, we compared IL-1β expression in BREC, RMC, RA, and RMG exposed to NG and HG. Under basal culture conditions (NG), IL-1β mRNA was detected in all four cell types examined. Exposure to HG for 4 days resulted in a significant upregulation of IL-1β in BREC (3 fold increase vs NG, *P* = 0.0001) (Fig. 1). The levels of IL1β expression in BREC exposed to equimolar mannitol or L-glucose were not different from those in control cells (NG), excluding increased osmolarity as the cause of the changes induced by HG. At difference with BREC, there was no effect of HG on IL-1β expression in Müller cells, astrocytes, and microglia neither after 4 days (Fig. 1) nor after longer exposure (7 days, not shown).

### Inhibition of PKC prevents the HG-induced upregulation of IL-1β in BREC

**Figure 2:**
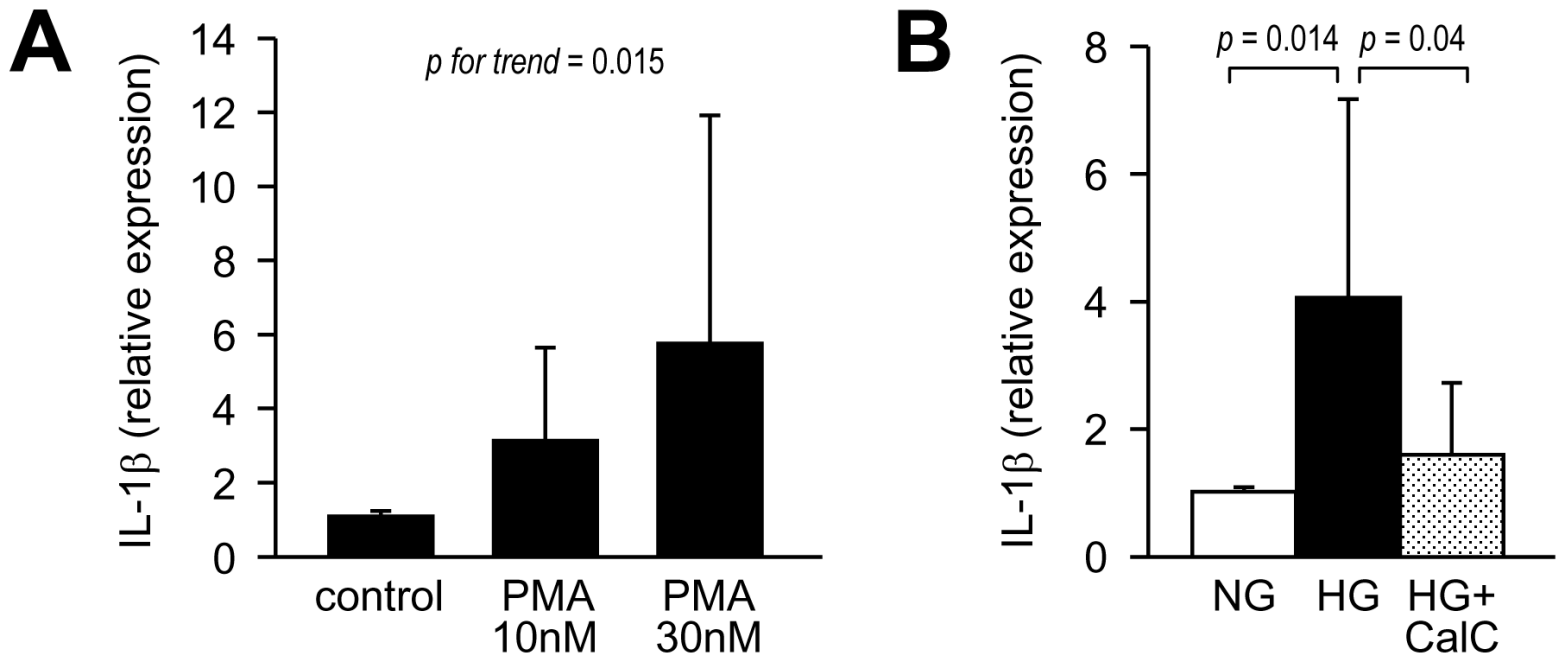
Effects of PKC inhibition on HG-induced IL-1β upregulation in BREC. (***A***) Effect of PKC activation on IL-1β expression. Confluent cultures of BREC (NG medium) were stimulated with PMA (10 and 30 nM) for 4 hours. Total RNA was extracted and IL-1β mRNA levels were assayed by quantitative RealTime RT-PCR. Bars represent mean ± SD of the results obtained in three different isolates tested in independent experiments. n = 9/group (***B***) Confluent cultures of BREC were exposed to NG, HG, or HG in the presence of the non-selective PKC inhibitors calphostin C (CalC, 50 mM) for 4 days. Total RNA was extracted and IL-1β mRNA levels were assayed by quantitative RealTime RT-PCR. Bars represent mean ± SD of the results obtained in three different isolates tested in independent experiments. n = 6/group.

Upregulation of IL-1β in both aortic and retinal endothelial cells has been described, but the mechanisms underlying the effect of HG are not known. In monocytes, protein kinase C (PKC) activation is sufficient to induce IL-1β expression [36] and upregulation of IL-1β by HG has been reported to occur via a PKC-dependent mechanism [24]. Thus, we studied whether PKC activation is sufficient to induce IL-1β expression in retinal endothelial cells, and whether this mediates the effect of HG on IL-1β expression in these cells. Stimulation of BREC with PMA led to a significant, dose-dependent upregulation of IL-1β (Fig. 2A). The degree of IL-1β induction by PMA (relative expression: 3.0±2.6 PMA 10 nM and 5.7±6.1 PMA 30 nM vs 1.0±0.2 in control), albeit variable among different isolates, was comparable to that of HG. Addition of calphostin C, a non-selective PKC inhibitor, to BREC cultured in HG prevented the HG-induced upregulation of IL-1β (relative expression: 1.6±1.1 in HG+calphostin C vs 4.1±3 in HG, *P* = 0.04, and 1.0±0.01 in NG, *P* = 0.6. HG vs NG, *P* = 0.014) (Fig. 2B). At the concentration used, calphostin C had no effect on cell viability and morphology (Fig. S2).

### IL-1β is upregulated in retinal vessels of diabetic rats

**Figure 3:**
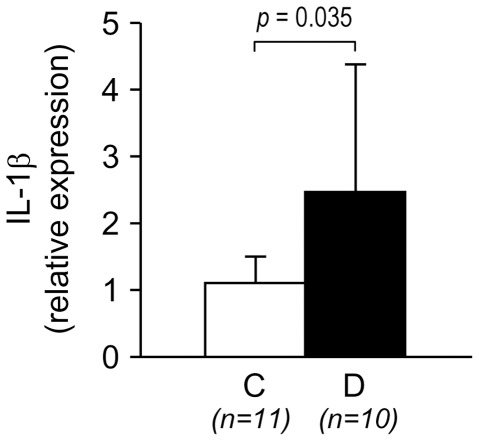
Upregulation of IL-1β in retinal vessels in diabetes. Retinal vessels were isolated by hypotonic lysis from diabetic rats (D, 5 months of diabetes duration) and age-matched control rats (C). IL-1β mRNA levels were quantified by RealTime RT-PCR. Bars represent mean ± SD of the results obtained in the indicated number of rats.

The finding that of the different cell types tested only retinal microvascular endothelial cells upregulated IL-1β when cultured in HG pointed to the retinal vascular endothelium as a primary source of IL-1β in diabetes. To test whether the retinal vessels are a source of IL-1β we examined IL-1β expression in isolated retinal vessels of diabetic rats and age-matched control rats. As shown in figure 3, IL-1β mRNA levels are significantly increased in the retinal vessels of diabetic rats as compared to control rat (2.5 fold vs NG, *P* = 0.035).

### IL-1β induces its own synthesis in BREC, Müller cells, and astrocytes; but not in microglia

**Figure 4:**
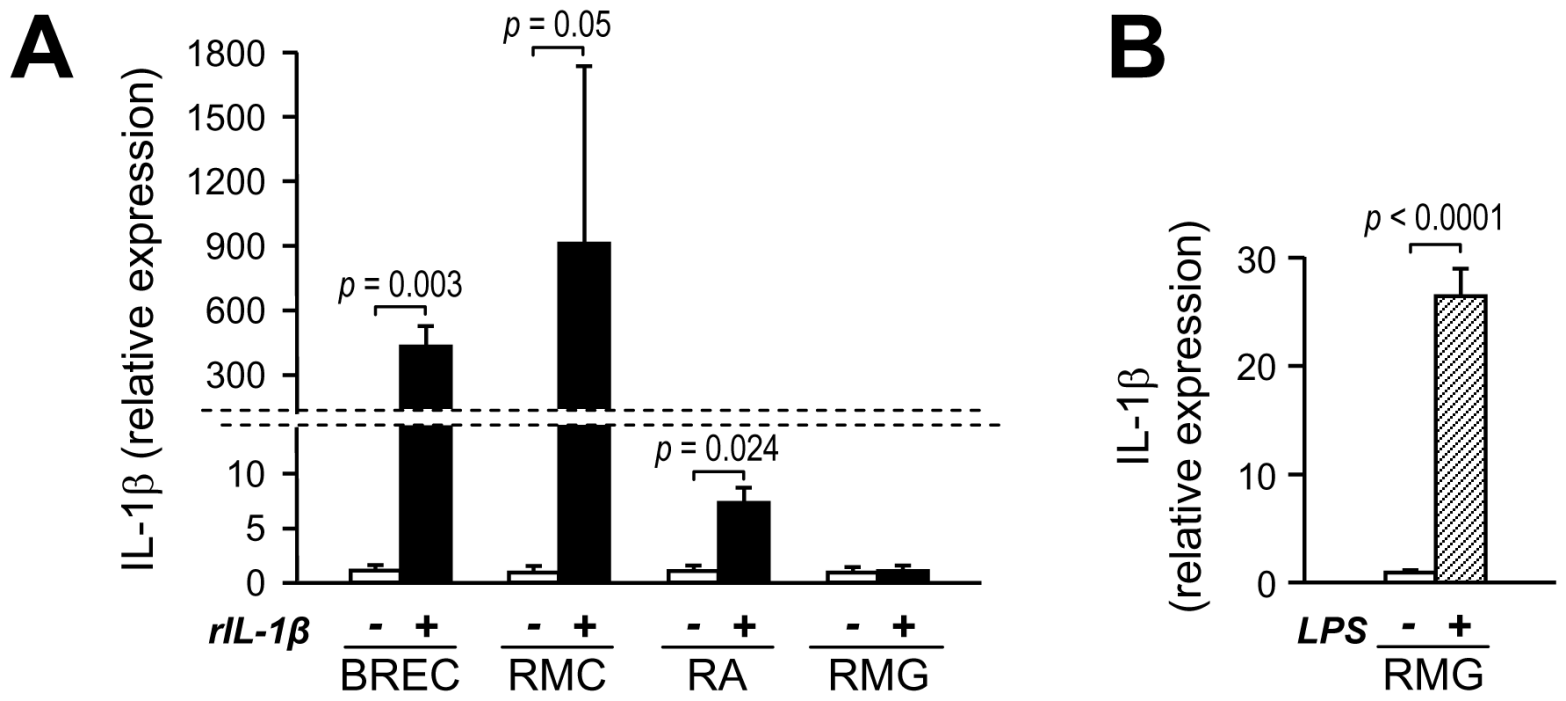
IL-1β autostimulation in retinal endothelial, macroglial, and microglial cells. (***A***) Effect of IL-1β on IL-1β expression. Confluent culture of BREC, RMC, RA, and RMG were pre-incubated in serum-free medium for 24 hours before stimulation with rIL-1β (bovine rIL-1β for BREC; human rIL-1β for RMC, RA and RMG) at 10 ng/ml of medium. Cells were harvested after 4 hours and IL-1β mRNA levels were assayed by quantitative RealTime RT-PCR. Bars represent mean ± SD of the results obtained from different isolates of each cell type. BREC, RMC, and RA: n = 3, RMG: n = 4. (***B***) Effect of LPS on IL-1β expression in microglia. RMG confluent cultures were pre-incubated in serum-free medium for 24 hours before stimulation with LPS at 1 µg/ml of medium for 4 hours. The expression of IL-1β was determined by RealTime RT-PCR. Bars represent mean ± SD of the results obtained from two isolates.

**Figure 5:**
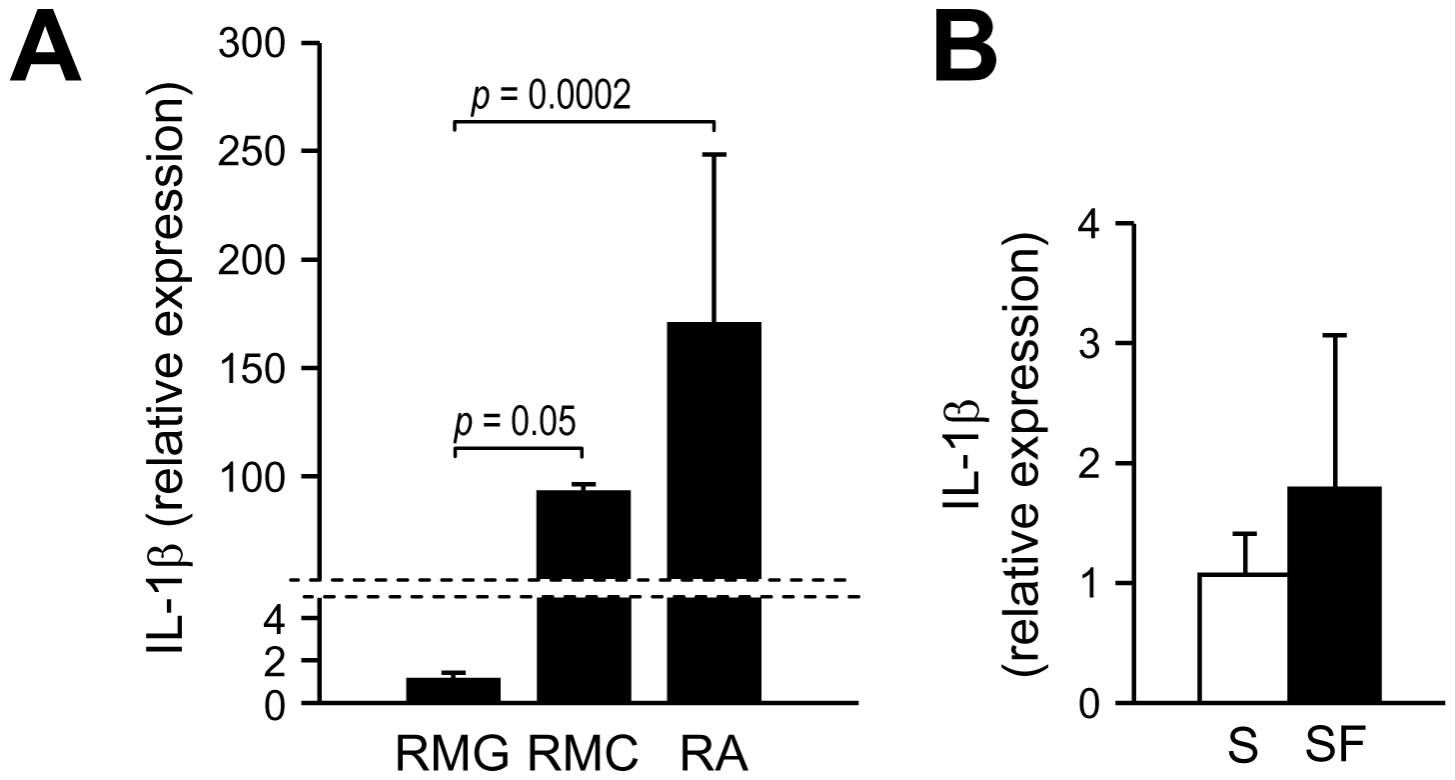
Differential expression of IL1R1 in rat microglial and macroglial cells. (***A***) Rat microglia (RGM), Müller cells (RMC), and astrocytes (RA) were cultured in NG medium for 4 days. Total RNA was extracted and IL1R1 mRNA levels were assayed by quantitative RealTime RT-PCR. IL1R1 expression in Müller cells and astrocytes was calculated relative to the expression in microglia by the comparative ΔΔC_T_ method using the mean ΔC_T_ obtained in microglial cells as the calibrator. Bars represent mean ± SD of the results obtained from 2–3 isolates per cell type. RMG: n = 6, RMC: n = 2, RA: n = 6. (***B***) IL1R1 expression in microglia cultured in serum-free medium. RMG, cultured in normal medium (10% FBS) (S) until confluency, were switched to serum-free medium (SF) for 24 hours before harvesting. Cells cultured in basal medium for the duration of the experiment were used as control. The expression of IL1R1 was determined by RealTime RT-PCR. Bars represent mean ± SD of the results obtained in three different isolates tested in independent experiments. n = 5/group.

IL-1β is known to induce its own synthesis via autocrine and paracrine autostimulation, a mechanism that magnifies upregulation of IL-1β expression. To test whether such mechanism could be operative in the diabetic retina, we measured the expression of IL-1β in BREC, RMC, RA, and RMG after stimulation with recombinant IL-1β. Treatment with IL-1β led to a significant upregulation of IL-1β in BREC (400 fold increase vs untreated, *P* = 0.003), Müller cells (900 fold increase vs untreated, *P* = 0.05), and astrocytes (7 fold increase vs untreated control, *P* = 0.024), but not in microglia (Fig. 4A). Because microglial cells failed to upregulate IL-1β in response to both high glucose and exogenous IL-1β, we used LPS to test the responsiveness of the cells to IL-1β-inducing stimuli. As shown in figure 4B, treatment with LPS led to a significant upregulation of IL-1β in the isolated microglia (26 fold increase vs untreated, *P*<0.0001). To determine whether the levels of expression of the IL-1β receptor could account for the different response of macroglial and microglial cells to IL-1β autostimulation, we compared the basal expression of IL1R1 in the different glial cell types. As shown in Fig. 5A although IL1R1 was detectable by quantitative RT-PCR in both microglia and macroglia, the levels of expression were ≥100 fold lower in microglial cells than in Müller cells or astrocytes. Pre-incubation of microglial cells in serum-free medium did not alter the expression of IL1R1 (Fig. 5B).

### Upregulation of IL-1β in the diabetic retina is associated temporally and quantitatively with glial activation

**Figure 6:**
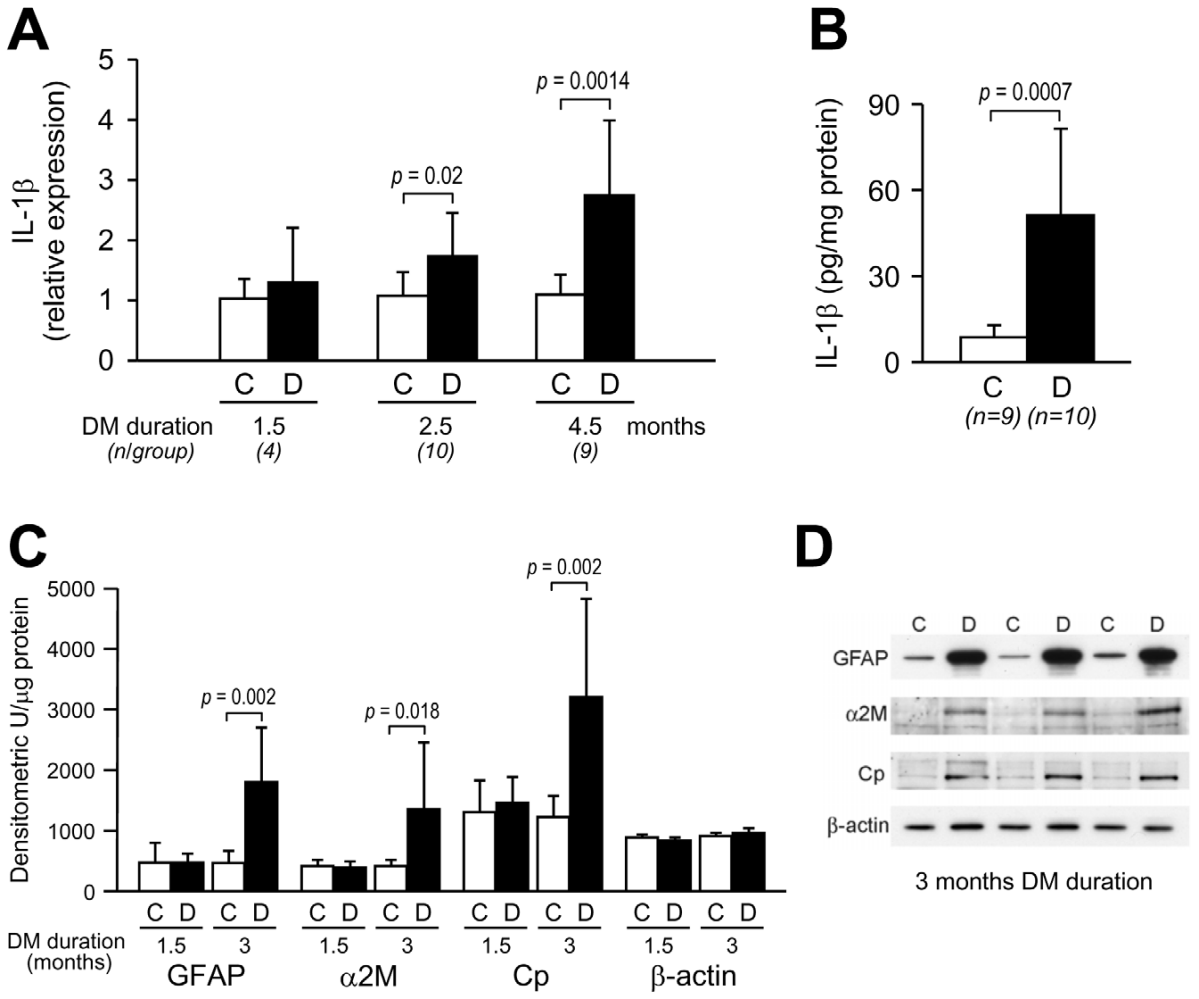
Effect of diabetes duration on the retinal expression of IL-1β and markers of glial reactivity. All experiments were conducted in diabetic (D) and age-matched control (C) rats. (***A***) Time course of IL-1β upregulation in relation to diabetes duration. IL-1β mRNA levels in the retina were quantified by RealTime RT-PCR. Bars represent mean ± SD of the results obtained in the indicated number of rats at each time point tested. (***B***) Increased protein levels of IL-1β protein in the retina in diabetes. IL-1β protein was quantified by ELISA at 5 months of diabetes duration. Bars represent mean ± SD of the results obtained in the indicated number of rats. (***C***) Time course of GFAP, α2-macroglobulin (α2M), and ceruloplasmin (Cp) expression. Protein levels were quantified by western blot analysis of whole retina lysates. Data are expressed as densitometric units per µg of protein. Bars represent mean ± SD. n = 4 rats/group at 1.5 months and 9 rats/group at 3 months. (***D***) Representative Western blots of GFAP, α2M, and Cp at 3 months of diabetes duration. β-actin was used as control for gel loading.

To learn about the possible relevance of IL-1β autostimulation in the diabetic retina in vivo, we studied the time course of IL-1β expression and its temporal relationship with the development of glial reactivity. IL-1β is a known inducer of glial activation. In the rat retina, IL-1β expression began to increase between 1.5 and 2.5 months after the onset of diabetes and continued to increase at later time points (Fig. 6A). At variance with IL-1β, the retinal expression of IL-6 and TNF-α was not changed in diabetes at any of the time points tested (Fig. S3). The increased transcription of IL-1β in the diabetic retina was associated with increased synthesis of the protein (6 fold vs control, *P* = 0.0007) (Fig. 6B). The time course of GFAP, α2-macroglobulin, and ceruloplasmin overexpression in the retina of diabetic rats was similar to that of IL-1β (Fig. 6C and 6D) with no changes as compared to control rats at 1.5 months from the induction of diabetes and a significant upregulation after 3 months of diabetes.

## Discussion

Our findings point to IL-1β as a trigger and propagator of retinal neuroinflammation in diabetes. This dual role of IL-1β is suggested by the in vitro observations that high glucose induces IL-1β in retinal vascular endothelial cells and IL-1β induces its own synthesis in retinal vascular endothelial cells, Müller cells, and astrocytes, combined with the in vivo observation that IL-1β upregulation in the diabetic retina increases progressively in relation to the duration of diabetes. Based on these data, we envision a scenario in which retinal upregulation of IL-1β in diabetes initiates in the vascular endothelium as a direct effect of hyperglycemia, and the secreted IL-1β stimulates in an autocrine and paracrine manner endothelial and macroglia cells, which respond not only with signs of activation but also with enhanced synthesis of IL-1β, thereby magnifying the features of neuroinflammation.

The high glucose-induced upregulation of IL-1β mRNA in retinal vascular endothelial cells is consistent with the observation by Kowluru et al of increased IL-1β protein levels in BREC exposed to high glucose [26]. However, Busik et al did not observe any effect of high glucose on the activation of ICE/caspase-1 and secretion of IL-1β in HREC [28]. Although this finding indicates that high glucose alone is not sufficient to increase processing and secretion of IL-1β in HREC, IL-1β mRNA was not evaluated by those authors and, thus, an effect of high glucose on IL-1β synthesis in HREC could not be excluded. Our finding of increased IL-1β expression in rat retinal vessels in diabetes indicates that retinal vascular cells are a source of IL-1β in vivo. While the analysis of isolated retinal vessels does not permit to discriminate between endothelial cells and pericytes/vascular smooth muscle cells as the source of IL-1β, the HG-induced upregulation of the cytokine in cultured endothelial cells proposes vascular endothelium as a cellular source of IL-1β overexpression in response to hyperglycemia.

The mechanisms of IL-1β induction in response to bacterial endotoxins or IL-1β itself are well characterized [37], but the signaling pathways linking high glucose to IL-1β upregulation are still poorly understood. The only available data are from studies of monocytes demonstrating that the induction of IL-1β by high glucose is mediated in these cells by a PKCα-dependent activation of p38MAPK, ERK, and NF-kB [24]. PKC activation has been shown to occur in retinal endothelial cells exposed to high glucose [38] as well as in the diabetic retina [39], [40], and has been proposed as a mechanism underlying endothelial dysfunction in diabetes. Our findings of IL-1β upregulation by the PKC agonist PMA and prevention of the HG-induced IL-1β upregulation in BREC by calphostin C point to upregulation of IL-1β as a potential link between PKC activation and vascular dysfunction in diabetes.

In contrast to the effect on BREC, high glucose did not lead to upregulation of IL-1β in microglia and macroglia. In cerebral ischemia, brain vascular endothelial cells have been implicated as a primary source of IL-1β [41], but extensive evidence indicates that the initial cellular source of IL-1β in response to CNS injuries is activated microglia with subsequent upregulation of the cytokine in macroglia [13]. In the diabetic retina, microglia activation precedes the occurrence of neuronal death [7] and high glucose has been shown to induce IL-1β expression in monocytes [24]. These observations would suggest that microglial cells could contribute to the hyperglycemia-induced upregulation of IL-1β. However, our data, showing that high glucose is not sufficient to induce IL-1β overexpression in microglial cells, are against this hypothesis. One caveat in interpreting these results is that we performed the study using brain-derived microglial cells and, thus, we cannot exclude a direct effect of high glucose on IL-1β expression that is specific to the retinal microglia.

As in microglial cells, high glucose had no effect on IL-1β expression in astrocytes and in Müller cells. The latter finding is at variance with previous reports of increased secretion of IL-1β by retinal Müller cells exposed to high glucose [26], [27]. Although an effect of high glucose on IL-1β expression has not been previously studied in astrocytes, increased secretion of IL-1β has been described for retinal Müller cells [27], [28]. A possible explanation for the discrepancy between our finding and those reports is that we measured IL-1β mRNA levels after a prolonged exposure to high glucose, whereas previous studies measured IL-1β protein secretion after a short exposure to high glucose. Under such circumstances, high glucose could have accelerated the processing of a pre-existing pool of pro-IL1β without changes in gene expression. Indeed, processing of pro-IL-1β followed by secretion of the mature protein has been proposed as the mechanism for the rapid release of IL-1β independent of de novo protein synthesis following traumatic brain injuries [42].

IL-1β can induce his own synthesis in an autocrine and paracrine fashion, a mechanism that is thought to propagate and amplify the inflammatory response [43]. Indeed, IL-1β-induced IL-1β synthesis has been previously described in human macrovascular endothelial cells [14] and other cell types [15]–[18]. In this study, we found that IL-1β induces IL-1β expression also in retinal microvascular endothelial cells, Müller cells, and, albeit to a lesser extent, astrocytes. The IL-1β-mediated induction of IL-1β observed in BREC and Müller cells suggests that the glucose-mediated increase of IL-1β in retinal endothelial cells might lead to further increase of IL-1β in the diabetic retina by autocrine and paracrine self-amplification of IL-1β expression in endothelial and macroglial cells. The occurrence of an IL-1β-driven IL-1β overexpression in the diabetic retina is supported by the progressive, disease duration-dependent increase of IL-1β mRNA – from 1.6 fold at 2.5 months and 2.5 fold at 4.5 months in this study, to 10 folds at 6 months of diabetes in our previous study [10]– a temporal pattern of IL-1β upregulation consistent with a positive feed-back effect of IL-1β itself.

At variance with the observation in endothelial and macroglial cells, we did not find evidence of IL-1β autostimulation in microglia. In neuroinflammation, activated microglia is recognized as the major cellular source of IL-1β. However, whether microglial cells are a target of the cytokine and whether IL-1β induces its own synthesis in these cells remains unclear [44]. IL-1β has been shown to induce its own synthesis in human fetal microglial cells [45] but not in mouse neonatal microglia [46]. We have now found that, similarly to mouse microglia, rat microglial cells do not upregulate IL-1β in response to IL-1β, but do so when stimulated with LPS. Since IL1R1 and TLR4 – the LPS receptor – share the same downstream signaling molecules [47], IL-1β induction by LPS indicates that the IL1R1/TLR signaling pathway is functional in these cells and that the failure of IL-1β to induce IL-1β in rat microglia is most likely due to the low expression of IL1R1.

IL-1β induces gliosis in vivo [48] and, in vitro, stimulation of astrocytes with IL-1β leads to upregulation or de-novo synthesis of several of the genes known to be regulated in reactive glia [49]. Glial reactivity is a key component of neuroinflammation and retinal glial reactivity is a well documented early feature of both human and experimental diabetic retinopathy [5], [6]. Our previous investigation of the reactive phenotype of Müller cell in rats with 6 months of diabetes identified the upregulation/induction of α2-macroglobulin, ceruloplasmin, and other acute-phase proteins as a major component of Müller cells reactivity [10]. The upregulation of acute-phase proteins was associated with retinal upregulation of IL-1β, but not of TNF-α or IL-6 [10]. In this study, we observed that the retinal expression of GFAP and acute-phase proteins increases in relation to diabetes duration with a pattern similar to that of IL-1β upregulation. Similar to our previous study of rats with longer disease duration, we did not find changes in the retinal expression of TNF-α, which is in contrast with other reports in the literature. However, in two of those other reports [50], [7], the observation of increased retinal TNF-α in response to diabetes was made after 1 or 2 weeks of diabetes and such increase could have been secondary to the initial acute metabolic imbalance after the induction of diabetes or to streptozotocin toxicity. In other two reports [51], [52], the increase in TNF-α was found in rats with durations of diabetes similar to those of our studies. One possible explanation for the discrepancy between those results and ours is the treatment of diabetic rats. To prevent weight loss and permit weight gain while maintaining hyperglycemia, the animals in our studies were treated with small doses of insulin from the onset of diabetes, whereas it appears that the animals in those other studies were not insulin treated. While our finding does not prove causality between upregulation of IL-1β and macroglial activation, the temporal association between upregulation of acute-phase proteins and IL-1β without changes of IL-6 and TNF-α proposes IL-1β as a candidate mediator of glial activation in the diabetic retina.

In summary, our findings point to hyperglycemia as the trigger and to the vascular endothelium as the origin of the initial increase of IL-1β expression in the diabetic retina, and to IL-1β itself, via autocrine/paracrine autostimulation in endothelial and glial cells, as the mechanism of sustained retinal upregulation of this cytokine. Because of the role that IL-1β can have in the development of retinal glial activation and endothelial dysfunction in diabetes, interrupting the vicious circle triggered by IL-1β autostimulation could be a mechanism to limit the progression of diabetic retinopathy.

## Supporting Information

Table S1 Primers and probe sets for RealTime RT-PCR analysis of IL-1β in BREC.
(PDF)

Figure S1 Characterization of BREC, RMG, RA, and RMC isolates.
(PDF)

Figure S2 Calphostin C does not affect BREC viability.
(PDF)

Figure S3 Diabetes does not alter the expression of IL-6 and TNF-α in the retina.
(PDF)
