# Supplementary material for: IL-1β Is Upregulated in the Diabetic Retina and Retinal Vessels: Cell-Specific Effect of High Glucose and IL-1β Autostimulation
Source: PLoS One. 2012 May 16;7(5):e36949. doi: 10.1371/journal.pone.0036949 (PMC3353989; doi:10.1371/journal.pone.0036949)
Supplement: Table S1 — Primers and probe sets for RealTime RT-PCR analysis of IL-1β in BREC. (PDF) [file pone.0036949.s001.pdf]

Table S1. Primers and probe sets for RealTime RT-PCR analysis of IL-1β in BREC.

	Primers/probe set		Reference Sequence
bovine IL-1β	forward	5’-TCC ACC TCC TCT CAC AGG AAA-3’	NM_173979
	reverse	5’-CTC TCC TTG CAC AAA GCT CAT G-3’,	
	probe	5’FAM-CAC CAC TTC TCG GTT CA-MGB3’	
bovine β-actin	forward	5’-AAA TGC TTC TAG GCG GAC TGT TAG-3’	NM_174039
	reverse	5’-TTC TGC GCA AGT TAG GTT TTG TC-3’	
	probe	5’FAM-CTG CGT TAC ACC CTT T-MGB3’	
