# Supplementary material for: IL-1β Is Upregulated in the Diabetic Retina and Retinal Vessels: Cell-Specific Effect of High Glucose and IL-1β Autostimulation
Source: PLoS One. 2012 May 16;7(5):e36949. doi: 10.1371/journal.pone.0036949 (PMC3353989; doi:10.1371/journal.pone.0036949)
Supplement: Figure S1 — Characterization of BREC, RMG, RA, and RMC isolates. (PDF) [file pone.0036949.s002.pdf]

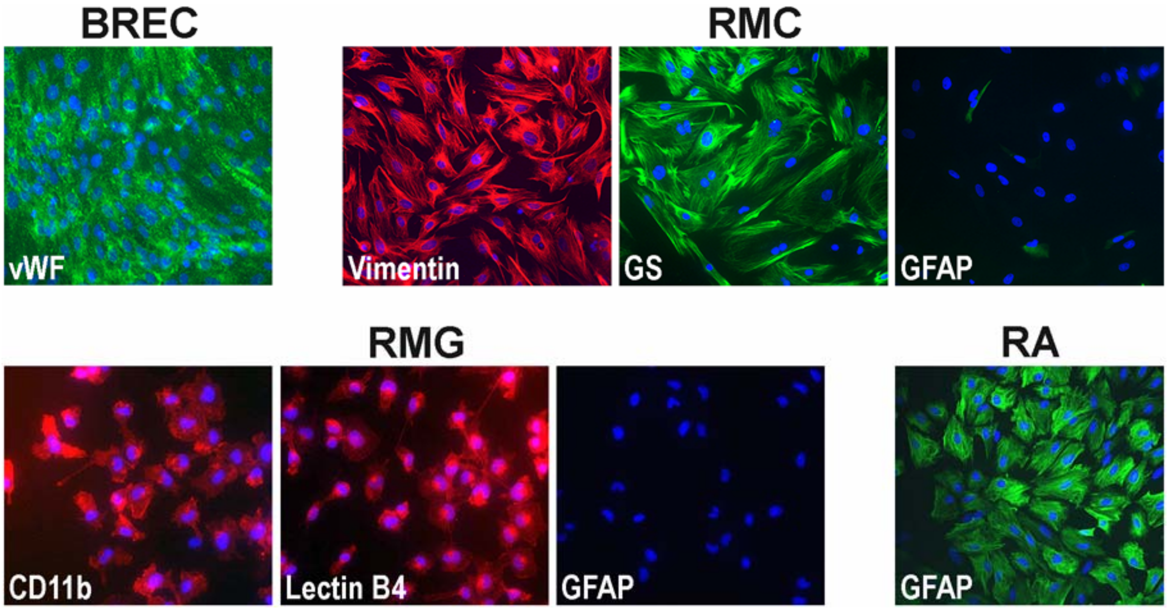

Figure S1. Characterization of bovine retinal endothelial cells (BREC) and rat Müller cells (RMC), microglia (RMG), and astrocytes (RA) isolates. Cells were isolated and cultured as described in Methods. Purity was assessed by immunostaining for cell-specific markers as described (Ref 10) using the following mouse monoclonal primary antibodies: anti-von Willebrand factor (vWF) (Dako) for endothelial cells; anti-vimentin clone V9 (Sigma) and anti-glutamine synthetase (GS) (Chemicon) for Müller cells; anti-CD11b (integrin alpha M/Mac1) clone OX-42 (Chemicon) for microglia. Microglial cells were further identified by isolectin B4 (Vector Laboratory) binding. A rabbit polyclonal anti-GFAP (Dako) antibody was used to detect astrocytes. Nuclei are visualized by DAPI counterstaining (blue). BREC cultures shows elongated spindle-shaped morphology and longitudinal alignment typical of endothelial cells. Purity is indicated by the homogeneous expression of vWF. Purity of RMC cultures is indicated by the characteristic elongated shape and the expression of vimentin and GS. The Müller cells origin is confirmed by the lack of GFAP positivity, consistent with the low levels of GFAP expression in non-activated Müller cells. Purity of RMG primary cultures is indicated by the amoeboid morphology, positive immunostaining for CD11b, and binding of isolectin B4. Microglial cultures are free of astrocytes contamination as indicated by the absence of GFAP positive cells. Purity of RA cultures is indicated by the expression of GFAP and the epithelial-like morphology characteristic of cultured astrocytes.
