# Supplementary material for: IL-1β Is Upregulated in the Diabetic Retina and Retinal Vessels: Cell-Specific Effect of High Glucose and IL-1β Autostimulation
Source: PLoS One. 2012 May 16;7(5):e36949. doi: 10.1371/journal.pone.0036949 (PMC3353989; doi:10.1371/journal.pone.0036949)
Supplement: Figure S2 — Calphostin C does not affect BREC viability. (PDF) [file pone.0036949.s003.pdf]

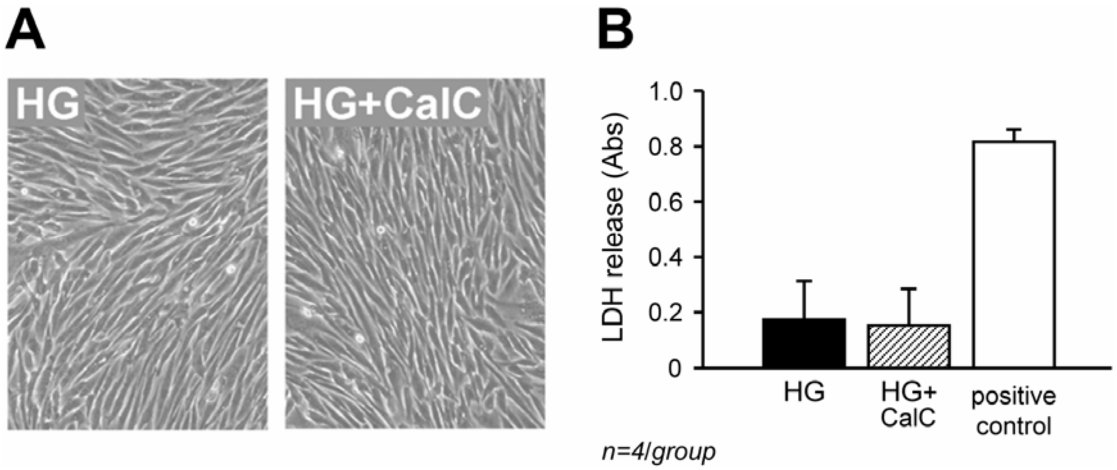

Figure S2. Calphostin C does not affect BREC viability. BREC were cultured as described in Methods. Cells death was determined by the LDH cytotoxicity assay (CytoTox 96 Assay, Promega). **(A)** Representative phase contrast microphotographs of confluent BREC monolayers after 4 days exposure to either HG or HG+calphostin C (CalC). **(B)** Bar plot of LDH release. Values are mean ± SD of the data obtained in three different isolates.
