# Supplementary material for: IL-1β Is Upregulated in the Diabetic Retina and Retinal Vessels: Cell-Specific Effect of High Glucose and IL-1β Autostimulation
Source: PLoS One. 2012 May 16;7(5):e36949. doi: 10.1371/journal.pone.0036949 (PMC3353989; doi:10.1371/journal.pone.0036949)
Supplement: Figure S3 — Diabetes does not alter the expression of IL-6 and TNF-α in the retina. (PDF) [file pone.0036949.s004.pdf]

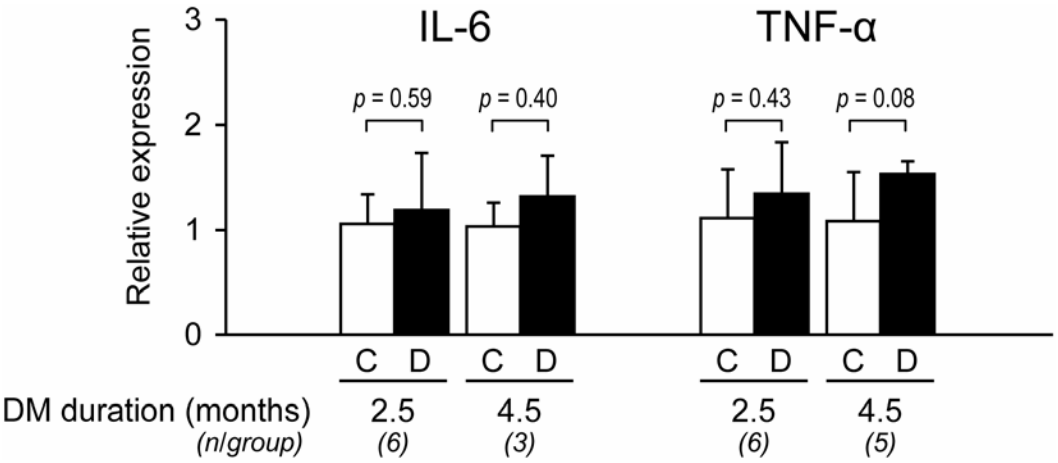


**Figure S3. Diabetes does not alter the expression of IL-6 and TNF-α in the retina.** All experiments were conducted in STZ-diabetic (D) and age-matched control (C) rats. IL-6 and TNF-α mRNA levels were quantified by RealTime RT-PCR as described in Methods. Bars represent mean ± SD of the results obtained in the indicated number of rats at each time point tested.
